# Supplementary material for: Content-rich biological network constructed by mining PubMed abstracts
Source: BMC Bioinformatics. 2004 Oct 8;5:147. doi: 10.1186/1471-2105-5-147 (PMC528731; doi:10.1186/1471-2105-5-147)
Supplement: Additional File 2 — The original results of the above study (non-essential files are deleted to keep the file size under the limit set by BMC bioinformatics). [file 1471-2105-5-147-S2.bz2 › chilibotAdditionalFile2/dip50/56ID8493578E211/html/RB1_E7.html]

 


 **RB1** and **E7** 
  
Found 222 abstracts in PubMed, retrieved 50.  
 

 What does Google say? 
 PDF only 
| .edu only 

---

**Interactive relationship** (e.g. stimulation, inhibition, etc)

**Inhibitory relationship**- The human papillomavirus HPV type 16  **E7**  oncoprotein must inactivate the retinoblastoma  [ **RB1** ]  tumor suppressor Rb pathway to bypass G 1 arrest.  Ref: 12239337 J Virol, 2002
- The transforming activity of  **E7**  has been attributed in part to its interaction with the retinoblastoma  [ **RB1** ]  Rb tumour suppressor.  Ref: 11753645 Oncogene, 2001
- Degradation of the retinoblastoma  [ **RB1** ]  tumor suppressor by the human papillomavirus type 16  **E7**  oncoprotein is important for functional inactivation and is separable from proteasomal degradation of  **E7** .  Ref: 11462030 J Virol, 2001
- These properties of  **E7**  depend on its ability to inhibit the activity of retinoblastoma  [ **RB1** ]  protein pRB, which in turn affects E2F function.  Ref: 11713253 J Biol Chem, 2002
- The retinoblastoma  [ **RB1** ]  protein pRb, the gene product of the first reported tumour suppressor gene, is functionally inactivated by the  **E7**  protein of high risk human papillomavirus HPV found in most human cervical cancers.  Ref: 11720846 Eur J Cancer, 2001
- HPV 18  **E7**  promotes S phase re entry in post mitotic differentiated keratinocytes by abrogating the transcription repression of E2F transcription factors by the retinoblastoma  [ **RB1** ]  susceptibility protein, pRb.  Ref: 11877408 J Biol Chem, 2002
- Human papillomavirus 16  **E7**  HPV16  **E7**  and adenovirus 5 E1A Ad5 E1A are encoded by highly divergent viruses yet are functionally similar in their ability to bind the retinoblastoma  [ **RB1** ]  pRB tumor suppressor protein, causing the aberrant displacement of E2F trancription factors.  Ref: 11123931 Biochemistry, 2000
**Neutral relationship**- Most of them express E6 and  **E7**  oncoproteins, able to bind to p53 and retinoblastoma  [ **RB1** ]  pRb tumor suppressor proteins respectively and neutralize their function.  Ref: 11948417 Oncogene, 2002
- Among these is the retinoblastoma  [ **RB1** ]  gene product pRB, which is inactivated by  **E7** .  Ref: 11291057 Int J Cancer, 2001
- Human papillomavirus type 16  **E7**  binds to E2F1 and activates E2F1 driven transcription in a retinoblastoma  [ **RB1** ]  protein independent manner.  Ref: 11713253 J Biol Chem, 2002
- Human papillomavirus  **E7**  HPV  **E7**  is a viral oncoprotein that plays an important role in cervical carcinogenesis through binding with retinoblastoma  [ **RB1** ]  protein Rb.  Ref: 12526093 Exp Mol Med, 2002
- Expression of the human papillomavirus type 16 E6 and  **E7**  oncogenes initiates and maintains abnormal cell replication, by interacting with the p53 and retinoblastoma  [ **RB1** ]  Rb gene products.  Ref: 12386813 Oncogene, 2002
- Thus, the similarities and differences in comparison to E1A, T antigen, and  **E7**  make pp71 an interesting new tool with which to further dissect the role of the retinoblastoma  [ **RB1** ]  E2F pathway in cellular growth control and carcinogenesis.  Ref: 12612064 Mol Cell Biol, 2003
- The HPV oncoproteins E6 and  **E7**  are thought to play a crucial role in this process through their interactions with the p53 protein and the retinoblastoma  [ **RB1** ]  susceptibility gene product pRb, respectively.  Ref: 11378660 Braz J Med Biol Res, 2001
- **E7**  oncoproteins of mucosal high risk human papillomavirus type 16 and 18 HPV16 and HPV18 immortalize primary rodent cells and transform them in collaboration with the activated ras, possibly by interaction with retinoblastoma  [ **RB1** ]  gene product RB and its related p107.  Ref: 11764290 J Investig Dermatol Symp Proc, 2001
- The steady state level and metabolic half life of retinoblastoma  [ **RB1** ]  tumor suppressor protein pRB are decreased in cells that express high risk human papillomavirus HPV  **E7**  proteins.  Ref: 11462030 J Virol, 2001
- Expression of the HPV E6 and  **E7**  oncoproteins increases in differentiating keratinocytes, resulting in inactivation of the p53 and retinoblastoma  [ **RB1** ]  proteins, two important transcriptional regulators.  Ref: 11287578 J Virol, 2001

**Non-interactive relationship** (e.g. studied together, co-existance, homology, etc.)

- Results obtained on fibroblasts retrovirally transfected with the human papillomavirus  **E7**  cDNA suggest that retinoblastoma  [ **RB1** ]  protein regulates the expression of TGF beta1 in stressful conditions, leading to SIPS and overexpression of these four genes.  Ref: 11060295 J Biol Chem, 2001
- Inactivation of both the retinoblastoma  [ **RB1** ]  tumor suppressor and p21 by the human papillomavirus type 16  **E7**  oncoprotein is necessary to inhibit cell cycle arrest in human epithelial cells.  Ref: 12239337 J Virol, 2002
- It has previously been shown that the  **E7**  protein from the cutaneous human papillomavirus type 1 HPV1, which is associated with benign skin lesions, binds the product of the tumor suppressor gene retinoblastoma  [ **RB1** ]  pRb with an efficiency similar to that of the  **E7**  protein from the oncogenic HPV type 16.  Ref: 11312342 J Virol, 2001
- In contrast, topo IIalpha expression is not affected by TGF beta1 in cells that fail to undergo G1 arrest because of inactivation of the retinoblastoma  [ **RB1** ]  tumor suppressor protein pRb by the papillomavirus type 16  **E7**  protein.  Ref: 11156401 Cancer Res, 2000
- E6 and  **E7**  target important cellular growth regulatory circuits among them the p53 and retinoblastoma  [ **RB1** ]  tumor suppressors, respectively.  Ref: 11861215 Front Biosci, 2002
- Diffuse, strong positivity with p16 in endocervical adenocarcinomas is likely caused by inactivation of the retinoblastoma  [ **RB1** ]  protein by the  **E7**  human papillomavirus oncoprotein, which acts as a p16 transcript repressor.  Ref: 12819388 Int J Gynecol Pathol, 2003
- Surprisingly,  **E7**  expression in terminally differentiated myotubes could not reactivate DNA synthesis even though the oncogene bound the retinoblastoma  [ **RB1** ]  protein, reduced its levels, and increased E2F transcriptional activity.  Ref: 12821937 Oncogene, 2003
- Binding to the retinoblastoma  [ **RB1** ]  tumor repressor Rb through an LxCxE motif in conserved region 2 is necessary, but not sufficient, for  **E7**  to induce proliferation.  Ref: 11435552 J Virol, 2001
- The high risk HPVs synthesize E6 and  **E7**  oncoproteins to alter the function of cellular regulatory proteins, such as p53 and retinoblastoma  [ **RB1** ]  gene product, respectively.  Ref: 12670501 Biochem Biophys Res Commun, 2003
- Destabilization of the retinoblastoma  [ **RB1** ]  tumor suppressor by human papillomavirus type 16  **E7**  is not sufficient to overcome cell cycle arrest in human keratinocytes.  Ref: 11435552 J Virol, 2001
- The HPV16 transforming proteins  **E7**  and E6 complex with protein products of Rb 1  [ **RB1** ]  and p53, respectively.  Ref: 11576229 Int J Gynecol Cancer< MedlineTA>Int J Gynecol Cancer, 2001
- Oligomerization properties of the viral oncoproteins adenovirus E1A and human papillomavirus  **E7**  and their complexes with the retinoblastoma  [ **RB1** ]  protein.  Ref: 11123931 Biochemistry, 2000
- Using defined mutants of  **E7**  and E1A, we show that an intact retinoblastoma  [ **RB1** ]  interaction domain is required for this function.  Ref: 11060027 EMBO J, 2000
- The p110 Rb protein level was also not detectable consistent with the expression of  **E7**  protein and haploid loss of  **Rb1**  gene.  Ref: 11073822 Am J Pathol, 2000
- The oncoproteins of the DNA tumor viruses, adenovirus E1A, simian virus 40 T antigen, and papillomavirus  **E7** , each interact with the retinoblastoma  [ **RB1** ]  family of tumor suppressors, leading to cell cycle stimulation, apoptosis induction, and cellular transformation.  Ref: 12612064 Mol Cell Biol, 2003
- Repression of the human papillomavirus HPV type 18 E6  **E7**  genes in HeLa cells by the bovine papillomavirus E2 transcriptional regulatory protein results in reactivation of the dormant p53 and p105 Rb  [ **RB1** ]  tumor suppressor pathways in these cells, repression of telomerase, and profound growth arrest.  Ref: 11714633 Cell Growth Differ, 2001
